# Asthma and gastroesophageal reflux: End of the blame game

**DOI:** 10.4103/0970-2113.56340

**Published:** 2009

**Authors:** Sachin Kumar, Dheeraj Gupta

**Affiliations:** *Department of Pulmonary Medicine, Postgraduate Institute of Medical Educations and Research, Chandigarh, India. E-mail: dheeraj@indiachest.org*

Gastroesophageal reflux and asthma, both of which are common conditions, often coexist in the same patient. Sir William Osler first observed the association between asthma and gastroesophageal reflux (GER).[1] Since then the effect of one condition on another has been investigated quite frequently, with varying results. GER is reported to be particularly prevalent among patients with asthma and many researchers suggest that it may be a predisposing factor for asthmatic episodes. Esophageal pH-monitoring studies have shown that 32% to 84% of individuals with asthma have abnormal acid reflux, and about half of patients with asthma who have reflux have no symptoms.[2] However, despite the presence of numerous studies, a cause-and-effect relationship between GER and bronchial asthma has not been established and the controversy as to whether GER causes bronchoconstriction or is simply a temporal association remains to be resolved.

The reflux theory suggests that symptoms of asthma are due to reflux of acid into the oesophagus followed by aspiration into the proximal airway.[3] On the other hand, the reflex theory suggests that distal esophageal acidification results in vagal stimulation and consequent bronchoconstriction, independent of airway microaspiration.[4] In addition, some of the medications used for asthma treatment can aggravate GER.[5]

Previous trials have had conflicting results regarding the beneficial effects of treatment with proton-pump inhibitors in patients with asthma who have frequent symptoms of GER disease. A systematic review of 12 small trials concluded that, although most of the studies showed that asthma-related outcomes were better when the patients were treated with proton-pump inhibitors.[6] However, analysis of the trials included shows that asthma was diagnosed based on symptoms and not using any objective criteria. Similarly, GER was diagnosed based on symptoms and not using ambulatory Ph monitoring or other objective tests.[7]

More recently, Littner and colleagues reported the results of a 6-month placebo-controlled trial involving 207 patients with moderate-to-severe asthma and definite symptoms of gastroesophageal reflux.[8] Treatment with 30 mg of lansoprazole twice daily did not improve the primary outcome of daily asthma symptoms, but it did result in a reduction in exacerbations and an improvement in asthma- related quality of life. The reduction in exacerbations was greatest among patients taking more than one class of medication for control of asthma.

Kiljander and colleagues[9] conducted a 3-strata, 24-week, multicenter, international trial involving patients with asthma who had nocturnal asthma symptoms, symptoms of GER, or both, and who were treated with 40 mg of esomeprazole twice daily. Overall, there was no efficacy in terms of daily peak expiratory flow rate, exacerbations, or asthma symptoms. However, in the stratum of 350 patients who had symptoms of both GER and nocturnal asthma, the peak expiratory flow rate improved, but there was no benefit with respect to FEV_1_, rescue-inhaler use, symptom scores, or nocturnal awakening. The esomeprazole-related improvement was most pronounced among patients who were taking long-acting beta-agonists.

In light of above studies, current guidelines recommend that physicians consider evaluating patients who have poorly controlled asthma, especially those with night-time symptoms, for GER disease. If GER is present, treatment recommendations include the use of a proton-pump inhibitor. However, patients with asthma who are receiving treatment for GER incur substantially higher diagnostic and treatment costs than do patients with asthma of similar severity who are not receiving treatment for this diagnosis.[10]

Individuals with asthma are particularly prone to asymptomatic GER. As mentioned earlier, about half of patients with asthma who have GER have no symptoms.[11] Whether proton-pump inhibitors improve asthma control in patients with minimal or no symptoms of GER is unknown, and whether objective measurement of acid reflux can be used to tailor treatment with proton-pump inhibitors to individual patients has not been established.[2]

Recently, in a parallel-group, double-blind trial, 412 participants with inadequately controlled asthma, despite treatment with inhaled corticosteroids, and with minimal or no symptoms of GER were randomly assigned to receive either 40 mg of esomeprazole twice a day or matching placebo and followed up for 24 weeks.[2] Ambulatory Ph monitoring was used to ascertain the presence or absence of GER in the participants. In this study, no treatment effect was observed with respect to individual components of the episodes of poor asthma control or with respect to secondary outcomes, including pulmonary function, airway reactivity, asthma control, symptom scores, nocturnal awakening or quality of life. The presence of GER, which was documented by pH monitoring in 40% of participants with minimal or no symptoms, did not identify a subgroup of patients that benefited from treatment with proton-pump inhibitors.

This study differs from previous trials in that it excluded patients who had symptoms of GER twice or more times per week. The rationale was that these patients already have an indication for acid-suppression treatment, irrespective of their asthma. In the study population, authors found no benefit from proton-pump inhibitors with respect to any primary or secondary asthma-related outcome measure.

Therefore, taken as a whole, the weight of evidence indicates that proton-pump inhibitors might improve the control of asthma in patients with moderate-to-severe persistent asthma having acid reflux symptoms. However, despite a high prevalence of asymptomatic GER among patients with poorly controlled asthma, treatment with proton-pump inhibitors does not improve asthma control. Asymptomatic GER is not a likely cause of poorly controlled asthma. Although widely practiced, proton-pump inhibitors should not be routinely prescribed for asthma symptoms if the patient does not have symptoms of GER.
